# Generic Medicine Pricing Policies in Europe: Current Status and Impact

**DOI:** 10.3390/ph3030471

**Published:** 2010-03-05

**Authors:** Pieter Dylst, Steven Simoens

**Affiliations:** Research Centre for Pharmaceutical Care and Pharmaco-economics, Katholieke Universiteit Leuven, O&N 2 P.O. Box 521, Herestraat 49, 3000 Leuven, Belgium; Email: steven.simoens@pharm.kuleuven.be

**Keywords:** pharmaceutical policy, generic medicines, pricing, Europe

## Abstract

Generic medicine pricing is an area of national responsibility of European Union countries. This article aims to present the current status and impact of generic medicine pricing policies in ambulatory care in Europe. The study conducts a literature review of policies relating to free-pricing systems, price-regulated systems, price differentiation, price competition and discounts, and tendering procedures; and a survey of European generic medicine pricing policies. Competition from Indian generic medicine manufacturers, European variation in generic medicine prices and competition between generic medicine manufacturers by discount suggest that the potential savings to health care payers and patients from generic medicines are not fully realized in Europe. One way of attaining these savings may be to move away from competition by discount to competition by price. Free-pricing systems may drive medicine prices downwards under specific conditions. In price-regulated systems, regulation may lower prices of originator and generic medicines, but may also remove incentives for additional price reductions beyond those imposed by regulation. To date, little is known about the current status and impact of tendering procedures for medicines in ambulatory care. In conclusion, the European experience suggests that there is not a single approach towards developing generic medicine pricing policies in Europe.

## 1. Introduction

Medicines have made a contribution to improving the health status of populations over time. A recent literature review concluded that medicines are among the most valuable forms of health care and are instrumental in treating various diseases more effectively [1]. Medicines are a major driver of the significant increase in life expectancy observed over the previous decades, although it is difficult to estimate the exact quantitative impact of medicines on population health.

The contribution of medicines to population health has been accompanied by rising pharmaceutical expenditure, thus putting pressure on health care expenditure. The countries of the Organisation for Economic Co-operation and Development witnessed an annual average growth in pharmaceutical expenditure per capita of 4.6% from 1995 to 2005 [2]. Growth in pharmaceutical expenditure exceeded the annual average rise in health expenditure per capita of 4% and the annual average economic growth of 2.2% during the same period. 

Expanding pharmaceutical expenditures, the introduction of expensive biotechnology medicines and orphan medicines, medicine price inflation, the growth in medication volume [3], the ageing population, the rising prevalence of chronic conditions and the increasing need for individualized treatments. All these trends, together with the current financial and economic crisis, are challenging society to accommodate the introduction of new and more effective medicines, whilst containing costs.

**Figure 1:**
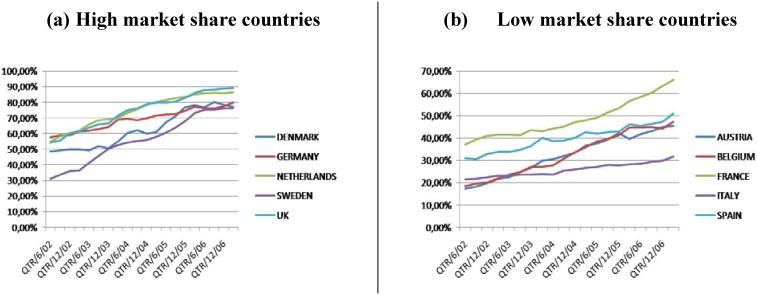
Evolution of generic medicine market share.

In the face of these pressures, a growing number of European countries pursue the development of their domestic generic medicines market. Generic medicines are substitutes for originator medicines with the same quality, safety and therapeutic efficacy. However, prices of generic medicines tend to be 10%–80% cheaper than those of originator medicines in Europe [4]. Due to their lower prices, generic medicines can yield significant savings for the pharmaceutical budget. Those savings could be used to provide headroom for innovation, giving health care payers the possibility to reimburse innovative and expensive new medicines [5]. Generic medicines market shares differ substantially in Europe. Figure 1 provides an overview of the evolution of the market shares in ten European countries from June 2002 until March 2007. Figure 1(a) shows the evolution in five countries with high generic medicine market share (Denmark, Germany, the Netherlands, Sweden and UK) while Figure 1(b) shows the evolution in five countries with low generic medicine market share (Austria, Belgium, France, Italy and Spain) [6]. Generic medicine market shares have increased in all ten countries and this is due to the various political measures taken by governments.

Pricing of generic medicines is an area of national responsibility of European Union member states. To date, little is known about how generic medicine pricing policies vary between countries, about the incentives that policies create for various stakeholders (e.g., industry, regulatory authorities, health care payers, physicians, pharmacists and patients), and about the impact that policies have on the objectives that countries wish to pursue. The European Commission recently provided an overview of pharmaceutical policies in European Union member states and examined the impact of these policies on such objectives as controlling pharmaceutical expenditure, ensuring patient access, rewarding innovation and maintaining pharmaceutical production [7]. However, the study did not focus specifically on generic medicine pricing policies.

The aim of this article is to present the current status and impact of generic medicine pricing policies in ambulatory care in Europe. To this effect, a literature review and a survey are carried out. The findings provide insight into policies and may serve to inform future policy options regarding pricing of generic medicines in Europe. It is however not the aim of this article to describe specific political measures in single countries. Other articles have already done this and provide a good overview [4,7,8,9]. Instead, this article derives lessons from the experiences of individual countries with generic medicine pricing policies.

## 2. Methods

The literature review identified studies by searching the following databases: Pubmed, Embase, National Health Service Economic Evaluation Database, Cochrane Database of Systematic Reviews, Web of Science and EconLit. The search strategy was developed using 14 combinations of ten search terms relevant to generic medicine pricing. The following ten search terms were used: pharmaceuticals, generic medicines, Europe, pricing, policies, regulation, competition, claw-back, discount and rebate.

Studies could be published in English, German, French, Dutch or Italian. The publication date was restricted to January 2000 until October 2009, as earlier studies were considered of limited relevance. The inclusion criteria were generic medicines, ambulatory care and European countries.

Once the relevant articles were selected, bibliographies of these articles were searched for additional references. Only peer reviewed articles were used and by using them, published information in the articles could already be dated as the investigated field is continuously evolving.

A survey was conducted to document the current status of generic medicine pricing policies in Europe. Survey data were collected from member associations of the European Generic Medicines Association in the context of their 2008 survey of European generic medicines retail markets [9]. The survey enquired about whether a country adopted a free-pricing system or a price-regulated system towards generic medicines. In the latter case, the approach to setting prices was elicited (e.g., an average price of selected European countries; a percentage below the price of the originator medicine; a maximum price; a negotiable price as a function of volume; or some other mechanism).

## 3. Results

### 3.1. Search Results

**Figure 2:**
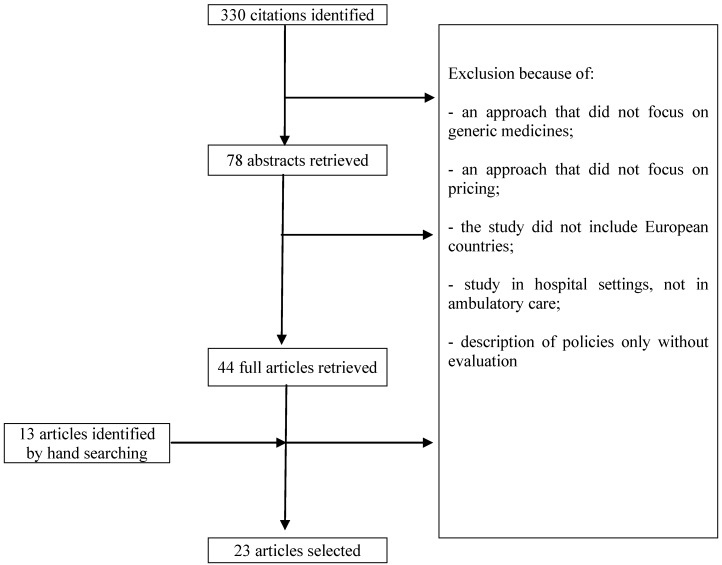
Flow chart of the literature search.

The literature search resulted in 688 citations: 256 with Pubmed, 165 with Embase, 21 with the Cochrane Database of Systematic Reviews, 66 with the National Health Service Economic Evaluation Database, 96 with Web of Science and 84 with EconLit. After the elimination of 358 duplicates, 330 articles were considered for inclusion. Figure 2 presents a flow chart of the literature search. In total, 320 articles were eliminated because of the following reasons: an approach that did not focus on generic medicines or pricing policies, a study that did not include European countries or ambulatory care, an article describing but not evaluating policies. Thirteen articles were added based on hand searching, so a total of 23 articles were selected for the literature review.

### 3.2. Price Differentiation

Generic medicine pricing varies around the world. The differentiation of prices can be seen at three levels: at international level, at the level of European countries and at the level of medicines within a specific country. 

Generic medicine pricing in Europe is influenced by price competition from, for example, Indian generic medicines manufacturers. Due to the benefits of low labour and production costs and weaker patent protection laws, India tends to produce generic medicines at lower prices than in Europe. A study compared prices of generic medicines of 15 molecule/strengths in ambulatory care in nine European countries and India in 2005. Generic medicines of these molecule/strengths were, on average, 63% cheaper in India than in the nine European countries [10]. As Europe is increasingly facing competition from India, this trend is likely to put downward pressure on European prices of generic (and originator) medicines. Indeed, Indian generic medicine prices could give an indication of the level to which generic medicine prices can drop in Europe in the future.

Several studies have compared ex-manufacturer prices of generic medicines between European countries [10,11]. Magazini *et al*. focused on generic medicines markets in the United Kingdom, Germany, Italy and France, and concluded that the dynamics of pricing following patent expiry varied significantly across countries [11]. A study which compared generic medicine prices in nine European countries observed the highest price levels in the United Kingdom, France, the Netherlands and Germany [8,10]. Higher prices in the United Kingdom, France and the Netherlands may derive from the fact that competition between generic medicines manufacturers in these countries takes the form of discounting to the distribution chain rather than price competition. Furthermore, the study indicated that generic medicine manufacturers do not use a single price setting strategy at European level. On the contrary, they tend to adapt their price setting strategy to the domestic regulatory environment surrounding registration, pricing, reimbursement and distribution of originator and generic medicines. Finally, this study pointed out that higher prices for generic medicines were observed in countries that had a developing generic medicines market (e.g., a generic medicine market share by volume below 40%). 

Generic medicine pricing not only varies between European countries, it also differs between medicines within a country. A study examined pricing strategies of originator and generic medicines following patent expiry in Belgium [8]. To this effect, the evolution of the public price of originator and generic medicines was investigated from July 2001 to December 2005. The analysis showed that pricing strategies in the Belgian off-patent market are influenced by regulatory aspects, such as successive reductions in reference prices and prescription status of medicines; by market incentives in the form of price competition between generic medicines, competition between originator and generic medicines or price agreements between originator and generic medicines; by medication class; and by the market power of the originator medicine. In other words, a unique pricing strategy that would predict the pricing behavior of originator and generic medicines did not exist.

### 3.3. Free-Pricing or Price-Regulated System

**Figure 3:**
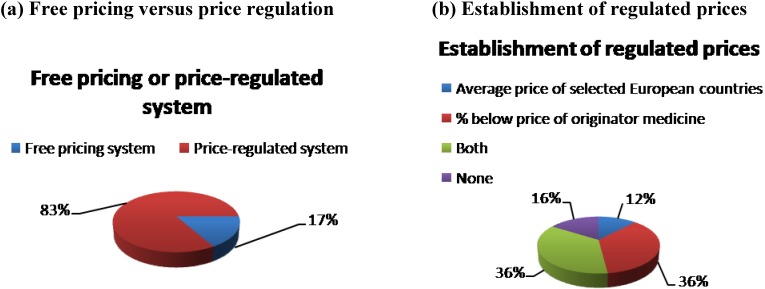
Generic medicine pricing systems in Europe, 2007.

European generic medicine pricing systems are either a free-pricing system, where manufacturers are (relatively) free to set generic medicine prices, or a price-regulated system, where generic medicine prices are set on a regulatory basis (e.g., by law). Figure 3 indicates that out a selection of 30 European countries in 2007, 83% of the countries had a price-regulated system whilst 17% had a free-pricing system [9]. Furthermore, in price-regulated systems, generic medicine prices were set as a percentage below the price of the originator medicine (36% of countries), as an average price of selected European countries (12% of countries), as a combination of both (36% of countries) or without any of them (16% of countries) (see Figure 3) [9].

The literature indicates that countries where manufacturers can price originator medicines freely have the highest prices, while countries with regulated systems have the lowest prices [12,13]. In countries where a free-pricing system is adopted, manufacturers of originator medicines can charge premium prices both before and after patent expiry. The high margins that can be earned attract market entry of generic medicines. In contrast, in countries that rely on regulated prices, regulation drives down the price of the originator medicine over the lifecycle of the medicine, thus discouraging market entry of generic medicines. The limited diffusion of generic medicines in such markets restricts price competition following patent expiry, although competition in the form of discounts to pharmacists may occur (e.g., France) [11,12,14,15].

The importance of the generic medicine pricing system was underlined by a study involving 11 European countries [4]. The authors found that penetration of generic medicines is more successful in countries that permit (relatively) free pricing of medicines than in countries that have price regulation. This may be because countries that adhere to free pricing generally have higher medicine prices, thereby facilitating market entry of generic medicines [12,13]. Also, in countries with free pricing, the price difference between originator and generic medicines tends to be higher than in countries with price regulation [16]. 

### 3.4. Price Regulation

Several mechanisms to regulate prices in pharmaceutical markets have been implemented, including price cap regulation and reference pricing. Under price cap regulation, the regulator sets a maximum price that can be charged for each medicine; whilst under reference pricing, the regulator establishes a common reimbursement level for a group of interchangeable medicines [17]. A study compared these two mechanisms in Norway. The results showed that pharmaceutical manufacturers respond differently to regulatory regimes. Reference pricing stimulated generic competition to a greater extent and led to lower prices than price cap regulation [17]. An unpublished literature review found that price cap regulation and reference pricing reduced prices of originator and generic medicines. At the same time, these mechanisms actually constituted a barrier for further price competition: there were no additional price reductions beyond those imposed by regulation [18].

### 3.5. Price Competition and Discounts

There is evidence that price competition by generic medicines lowers prices of originator medicines [15,19]. A study in Sweden pointed out that one additional generic competitor lowered prices by 4%–7% on average. Entrance of more generic competitors led to further reductions in prices of originator medicines [19]. The same phenomenon was observed in Italy [20]. However, competition does not take place on the basis of prices in all European countries. Instead, there is competition by discounts to wholesalers and to pharmacists in countries such as France and the United Kingdom. Two studies indicated that a discount of 10.74% is legally allowed in France but actual discounts vary from 20% to 70% of the wholesaler selling price [21], and discounts exceed 50% of the Drug Tariff Price in the United Kingdom [5]. 

Competition by discounts is not clear to market actors and is not fair as wholesalers and pharmacists are not rewarded for services rendered, but for their ability to negotiate discounts on artificial prices. Such a system may financially benefit wholesalers and pharmacists, but is not sustainable in the long run as health care payers and patients do not capture the potential savings from a generic medicines market where companies compete on price. As health care payers and patients are overpaying for medicines, some countries have taken additional measures. For instance, the United Kingdom has installed a claw-back mechanism to recover the discounts given to the pharmacists. However, this type of government intervention is unlikely to be as efficient as a market mechanism where manufacturers compete on the basis of prices rather than discounts to the distribution chain. Price competition between generic medicines manufacturers is transparent and easy for all market actors to understand, and ensures that prices paid by health care payers and patients reflect the actual value of the product.

### 3.6. Tendering

Tendering is a mechanism whereby a purchaser buys medicines from the pharmaceutical manufacturer that offers the best bid [22]. Whereas tendering procedures are widely used in the hospital sector, they are recently being rolled out in ambulatory care in an increasing number of countries with a view to constraining pharmaceutical expenditure. 

In Belgium, a tendering procedure has been applied to the medicine simvastatin. Data suggest that the simvastatin tender resulted in substantial price reductions and savings for the health care payer. However, this trend was counterbalanced by the growth in expenditures for other statins. Expenditure on medicines containing simvastatin decreased with 30% in 2008 versus 2007, while expenditure for medicines containing atorvastatin and rosuvastatin increased by 16% and 40%, respectively, in the same period. As a result, total expenditure on statins rose by 6.5%. The Belgian experience indicates that tendering produced savings for one specific medicine, but these savings were offset by the fact that physicians switched their prescribing patterns to medicines with a similar therapeutic indication that did not fall under the tendering procedure (so-called ‘re-allocation of demand’) [23].

In Germany, health insurance funds are able to engage in tendering procedures with pharmaceutical manufacturers of originator and generic medicines. Under the tender system, a patient receives a medicine with the active substance, dosage and pack size as prescribed by the physician from the specific manufacturer that has won the tender. Although no formal evaluation of the German tendering procedure has been undertaken, tendering appears to have resulted in a change in market share from larger to smaller pharmaceutical manufacturers and there have been reports of short-term absences of some medicines due to logistic shortages. 

A study assessed the economic impact of tendering procedures in some countries like Germany, the Netherlands and Denmark [24]. The results indicated that cost savings for the pharmaceutical budget were reached in the short term due to the significant reduction of medicines’ prices. However, a negative impact on the economy was observed in the long term. Countries that had implemented tendering procedures had witnessed a decrease in pharmaceutical investments. This had a negative impact on employment and caused a loss of income from taxes. The development and adoption of generic medicines was also slowed by tendering procedures. 

## 4. Discussion

Countries have taken an interest in generic medicine pricing policies as a means of, amongst other things, guaranteeing affordable access to medicines for their populations and of keeping pharmaceutical expenditure under control. 

There is no single approach towards developing generic medicine pricing policies in Europe. This paper has found that European countries have implemented a variety of generic medicine pricing policies, although there is little evidence of the current status and impact of generic medicine pricing policies in these countries. Furthermore, generic medicine pricing policies have grown incrementally in countries over time and reflect demographic, cultural, economic and institutional constraints. Therefore, there is no reference set of policy measures that countries can adopt with respect to generic medicine pricing.

With respect to generic medicine pricing, evidence has emerged that European generic medicine manufacturers face competition from Indian manufacturers; that the price level of generic medicines varies substantially between European countries; and that generic medicine manufacturers engage in competition by discount rather than price competition in countries such as France, the Netherlands and the United Kingdom. These trends suggest that the potential savings to health care payers and patients from generic medicines are not fully realized in Europe and that there may be scope for further reducing prices of generic medicines in several countries.

In the few European countries that have adopted a generic medicine free-pricing system, this has led to higher medicine prices, but also stimulated competition between generic medicines, thereby driving prices downwards. It seems that specific conditions need to be fulfilled for a free-pricing system to generate lower prices. The presence of competition seems to be essential as, without competition, pharmaceutical manufacturers have no incentive to lower their prices. Also, the ability of the generic medicines industry to offer competitive prices is likely to depend on the generic medicine market share. The European experience indicates that the generic medicines industry is able to deliver competitive prices if it is ensured a high volume of the pharmaceutical market [9]. This high volume is dependent on demand-side policies creating incentives for physicians, pharmacists and patients to ask for generic medicines.

The majority of European countries use a price-regulated system. Mechanisms such as price cap regulation and reference pricing have generated price reductions of originator and generic medicines, but appear to remove incentives for additional price reductions beyond those imposed by regulation. There may be a case for combining a free-pricing system with reference pricing. For instance, the common reimbursement level can be set at the average price level in the group of interchangeable medicines. In combination with incentives to stimulate demand for generic medicines, generic medicine manufacturers would have an incentive to compete, thereby driving down prices and reimbursement levels of medicines. Such a system may translate into greater price reductions than those that are imposed by regulation.

Information about generic medicine prices needs to be transparent. This may not be the case in countries where generic medicines manufacturers compete with each other through offering discounts to wholesalers and pharmacists. The practice of discounting is not clear to market actors, means that prices do not reflect the actual value of a medicine, and implies that health care payers and patients are overpaying for medicines. Therefore, countries need to consider moving away from competition by discount to competition by price. 

In addition to factors affecting the generic medicine price level in European countries, this paper has drawn attention to the determinants of generic medicine pricing within a country. The literature indicates that manufacturers of generic medicines do not adopt a single pricing strategy in a country, but take into account several factors, the importance of which may vary from molecule to molecule. As a result, there does not seem to be a single pricing model that authorities or manufacturers can use to predict the pricing behaviour of originator and generic medicines following patent expiry, and to foresee the development of the generic medicines market. Policy measures governing generic medicine pricing need to take into account this variety in pricing strategies.

Although many European countries are considering to implement or are implementing tendering procedures for medicines in ambulatory care, few data are available to date on the current status and impact of tendering procedures in Europe. There is a need for additional research to document European tendering procedures by exploring the objectives that are pursued by a tender, the legal basis surrounding a tender, the authorities in charge of a tender, the criteria used to award a tender, the frequency and duration of a tender, etc. Also, the impact of tendering procedures in the short term and the long term needs to be explored. Such research may provide further insight into tendering procedures and may inform future policy options regarding tendering procedures in Europe.

## 5. Conclusions

This paper has drawn attention to generic medicine pricing policies in Europe by focusing on free-pricing systems, price-regulated systems, price differentiation, price competition and discounts, and tendering procedures. In light of the European experience, there does not seem to be a single approach towards developing generic medicine pricing policies in Europe. Competition from Indian generic medicine manufacturers, European variation in generic medicine prices and competition between generic medicine manufacturers by discount suggest that the potential savings to health care payers and patients from generic medicines are not fully realized in Europe. One way of attaining these savings may be to move away from competition by discount to competition by price. It appears that free-pricing systems may drive medicine prices downwards under specific conditions. In price-regulated systems, regulation may lower prices of originator and generic medicines, but appears to remove incentives for additional price reductions beyond those imposed by regulation. To date, the current status and impact of tendering procedures for medicines in ambulatory care is unclear.
